# Spatiotemporal dynamics of the development of mouse olfactory system from prenatal to postnatal period

**DOI:** 10.3389/fnana.2023.1157224

**Published:** 2023-04-11

**Authors:** Bo-Ra Kim, Min-Seok Rha, Hyung-Ju Cho, Joo-Heon Yoon, Chang-Hoon Kim

**Affiliations:** ^1^Department of Medicine, Graduate School, Yonsei University, Seoul, Republic of Korea; ^2^Department of Otorhinolaryngology, Yonsei University College of Medicine, Seoul, Republic of Korea; ^3^The Airway Mucus Institute, Yonsei University College of Medicine, Seoul, Republic of Korea; ^4^Korea Mouse Sensory Phenotyping Center, Yonsei University College of Medicine, Seoul, Republic of Korea; ^5^Global Research Laboratory for Allergic Airway Diseases, Yonsei University College of Medicine, Seoul, Republic of Korea; ^6^Taste Research Center, Yonsei University College of Medicine, Seoul, Republic of Korea

**Keywords:** development, olfactory epithelium (OE), olfactory bulb (OB), olfactory sensory neuron (OSN), olfactory cilia, spatiotemporal

## Abstract

**Introduction:**

The olfactory epithelium (OE) and olfactory bulb (OB) are the major components of the olfactory system and play critical roles in olfactory perception. However, the embryonic development of OE and OB by using the olfactory specific genes has not been comprehensively investigated yet. Most previous studies were limited to a specific embryonic stage, and very little is known, till date, about the development of OE.

**Methods:**

The current study aimed to explore the development of mouse olfactory system by spatiotemporal analysis of the histological features by using the olfactory specific genes of olfactory system from the prenatal to postnatal period.

**Results:**

We found that OE is divided into endo-turbinate, ecto-turbinate, and vomeronasal organs, and that putative OB with putative main and accessory OB is formed in the early developmental stage. The OE and OB became multilayered in the later developmental stages, accompanied by the differentiation of olfactory neurons. Remarkably, we found the development of layers of olfactory cilia and differentiation of OE to progress dramatically after birth, suggesting that the exposure to air may facilitate the final development of OE.

**Discussion:**

Overall, the present study laid the groundwork for a better understanding of the spatial and temporal developmental events of the olfactory system.

## 1. Introduction

Olfactory epithelium (OE) and olfactory bulb (OB) are the major components of the olfactory system. Olfactory signals are generated by the binding of odorant molecules in air to the olfactory receptors (ORs) in the olfactory cilia of OE. The signals are transferred to the OB and subsequently to higher brain regions, such as the primary olfactory cortex. Therefore, OE and OB play critical roles in the initial perception and signal transduction of olfactory information.

The development of the mammalian olfactory system has been investigated in several previous studies by using histological and immunohistochemical methods. A few decades ago, the development was observed by using hematoxylin and eosin (H&E) staining, silver staining, electron microscopy, and radiographic method. Some groups used these histological methods, and their descriptions of the cytological and structural changes during the olfactory development using general histological organization provide a broad outline of olfactory development (Hinds, 1968; Cuschieri and Bannister, 1975a,b). According to these researches, differentiated neurons appeared and axons emerged at E10–10.5, dendritic formation appeared and axons reached the OB primordium at E11, the presumptive ONL (olfactory nerve layer) which consists of olfactory axons, glia cells, and several junction molecules appeared at E11.5–13.5, few cilia formed on the end of olfactory sensory neuron (OSN) at E12–16, the differentiated neurons in OE and the proliferation of basal stem cells appeared at E13–15, the maturation of differentiated neurons increased and stem cells remained at E17, and Bowman’s glands were visible at E17. In OB, formed cells differed mainly at the developmental stages: mitral cells at E10–12, tufted cells at E13–18, granule cells at E12–E18, and neuroglia precursors at E17-PN10.

Afterward, the studies using rodents carrying the mutation of a specific gene and staining with the specific gene revealed what role the specific gene plays during olfactory development. In the nasal forebrain junction (NFJ), migratory mass (MM) the other name, which appeared in early development, there are nerves (terminal nerves, olfactory nerves, and vomeronasal nerves), OEC (the olfactory ensheathing) cells, several signal molecules, and adhesion molecules (Miller et al., 2010; Taroc et al., 2017; Perera et al., 2020; Duittoz et al., 2022). For forming the hypothalamic–gonadal axis, GnRH (gonadotropin-releasing hormone expressing) neurons arose at E10.5–11.5 and migrated from vomeronasal organs (VNO) to the basal region of the brain, and gonadotropic axis formed (Forni and Wray, 2012, 2015; Duittoz et al., 2022). The anatomical location of OSNs expressed by distinct olfactory receptors correlated with their projection to the topographic region of OB. This determination of the specific region is defined by the pattern of several signal molecules like axon guidance molecules and distinct olfactory receptors expressed in each OSN. After the first olfactory receptor appeared at E11, the cilia prolonged and became diverse (Schwarzenbacher et al., 2004; McClintock et al., 2020). After placode formation, the OE structure deepened and complexed as the development progressed (Duggan et al., 2008; Chen et al., 2009). In OB, the development started from the projection of OSN axons, and the later phase progressed by the migration and localization of its neurons and glia (Gong and Shipley, 1995; Tufo et al., 2022). The glomeruli where olfactory axons and periglomerular neurons interact formed after birth (McLean and Shipley, 1988; Bastianelli and Pochet, 1995; Saino-Saito et al., 2004; Tufo et al., 2022). Also, OB got divided into MOB (main OB) projected by OSN axons in OE and AOB (accessory OB) projected by vomeronasal sensory neurons (VSN) axons in VNO, and then each layer differently differentiated during development (Martín-López et al., 2012). Through this overview of previous studies in olfactory development, the developmental relationship shows the link between OE and OB.

In these investigations, only part of the events in the development of olfactory system have been studied, compared to the continuous stages of development. For this reason, the time when the specific structure appeared differed in the previous studies. Moreover, very little is known about the change of cellular composition in OE during development. Also, timing of amplifying neurogenesis in OE, how OE and OB interact with each other, and what is the corresponding event of the first after birth encounter with the air is questionable. Therefore, a comprehensive analysis of the embryonic development of the olfactory system, by staining with the olfactory-specific markers, is imperative. In this study, we aimed to reveal the events in the mouse olfactory system during development.

## 2. Materials and methods

### 2.1. Mice

C57BL6/N mice from ORIENTBIO (breeding facility, Gyeonggi-do City, South Korea) were used in the present study. All mice were handled in strict accordance with the guidelines for the Care and Use of Laboratory Animals of Yonsei University College of Medicine, and the study was performed under the Yonsei Medical Center Animal Research Guidelines (IACUC No. 2020-0030), which adhere to the standards articulated in the Association for Assessment and Accreditation of Laboratory Animal Care International (AAALAC) guidelines.

### 2.2. Mating and plug check

After mating at the start of the night cycle (8 p.m.), we checked the plugs in virgin female mice at the start of the day cycle (8 a.m.). If the plugs were shown in the vagina of female mice, the day was considered as embryonic day 0.5 (E0.5). We used mice at E10.5–16.5, E18.5, postnatal day 1 (PN 1), 1-, 2-, and 4 weeks of age for the subsequent experiments.

### 2.3. Tissue treatment and sectioning

The nasal cavity of mice is mainly composed of flexible cartilage. After fixation of nasal cartilage tissues with 4% PFA (perfluoroalkoxy alkanes), decalcification was required to slice the tissues with flexible cartilage (from E14.5) in 10% EDTA (ethylenediaminetetraacetic acid) solution, pH 7.0, for 1 day–2 weeks as the thickness of tissues. Four-micron-thick paraffin sections and 10–15 μm-thick cryosections were used.

### 2.4. Immunostaining

Immunostaining in the present study was performed as follows. After removal of the matrix [paraffin or OCT (optimal cutting temperature) compound], the slides were boiled with antigen retrieval solution (IHC world, #IW-1100) in a steaming bowl for 10–40 min. After cooling, they were incubated in 3% hydrogen peroxide for 10 min. After washing with tris-buffered saline (TBS), the slides were blocked with 5% bis(trimethylsilyl)acetamide (BSA) for 1 h and incubated with primary antibodies for 1 h at 37°C or overnight at 4°C. We used rabbit anti-Sox2 [sex-determining region Y (SRY)-related high mobility group (HMG)-box] (Abcam, Cambridge, UK, 1:50), mouse anti-Tuj1 (Abcam, Cambridge, UK, 1:500), goat anti-OMP (olfactory marker protein) (Wako, Richmond, VA, USA, 1:200), rabbit anti-K5 (Keratin 5) (Abcam, Cambridge, UK, 1:250), rabbit anti-Golf (1:100), goat anti-ACIII (Adenylate Cyclase III) (Santa Cruz, Dallas, TX, USA, 1:50), rabbit anti-ERMN (Thermo Fisher Scientific, Waltham, MA, USA, 1:500), goat anti- Nrp2 (Neuropilin 2) (R&D systems, Minneapolis, MN, USA, 1:50), rabbit anti-TH (tyrosine hydroxylase) (Millipore, Burlington, MA, USA, 1:200), and mouse anti-Reelin (Millipore, Burlington, MA, USA, 1:50) as primary antibodies. After washing with TBS, the slides were blotted with fluorescent secondary antibodies from Thermo Fisher Scientific (Waltham, MA, USA) for 30 min at room temperature. After a final wash with TBS, the slides were mounted using a fluorescent mounting medium with 4′,6-diamidino-2-phenylindole (DAPI) (Sigma, St. Louis, MO, USA).

### 2.5. *In situ* hybridization

Only cryo-sectioned slides were used for *in situ* hybridization (ISH) in the present study. Before staining, the slides were air-dried to prevent the detachment of tissues from the slide glass during staining. After washing with polybutylene terephthalate (PBT), they were bleached with 5% H_2_O_2_ solution for 5 min. After another wash, the slides were incubated with 10 μM/mL Proteinase K for 1–5 min. After washing again, they were fixed with fixation solution (4% PFA, 0.25% glutaraldehyde) for 10 min. Following a final wash, they were blotted overnight with 1 μg/mL probes at 69°C.

The next day, for stabilizing the probes blotted on samples, the slides were incubated at 69°C, with a pre-warmed solution of high concentration of saline-sodium citrate (SSC) [50% ionized formamide, 6 × SSC, and 1% sodium dodecyl sulfate (SDS) diluted in sterile distilled water] thrice for 15 min each, and with a low concentration of SSC (50% ionized formamide and 2.4 × SSC diluted in sterile distilled water) thrice for 15 min each. After washing with tris-buffered saline with 0.1% Tween^®^ 20 detergent (TBST), they were blocked using blocking solution (5% inactivated sheep serum) for 1 h. Next, they were blotted with anti-digoxigenin antibody (Roche, Basel, Switzerland, 1:1000) at 4°C overnight. The next day, after washing with TBST, they were wrapped in foil to protect them from light. After washing with NTMT solution (20 mM NaCl, 5% of 2 M Tris HCl, pH 9.5, 10 mM MgCl_2_, 0.01% Tween 20, and 2 mM levamisole diluted in sterile distilled water) thrice for 30 min each, they were incubated with NTMT solution containing 250 μg/mL nitro blue tetrazolium (NBT) (Roche, Basel, Swiss) and 130 μg/mL 5-bromo-4-chloro-3-indolyl phosphate (BCIP) (Roche, Basel, Swiss) until the desired stain intensity developed within 1–2 days. The reaction for color formation was stopped using 1 mM EDTA, diluted with PBT, for 30 min. Finally, the slides were mounted.

The ISH probes were prepared as follows: using the RNA samples extracted from the olfactory epithelium of an embryo or adult as the template, partial cDNA of achaete-scute homolog 1 ASCL1 was amplified by PCR (polymerase chain reaction). The specific primers used for ASCL1, including a sequence of T7R polymerase were forward primer: 5′-TCGTCCTCTCCGGAACTGAT-3′ and reverse primer: 5′-TAATACGACTCACTATAGGGAGACCCATCTGTGATTCGGG CTT-3′ (Ensemble Accession No: NM_008553.5). Using the PCR products as templates, the tubes with 20 U ribonuclease inhibitors (Promega, Madison, WI, USA), 1 × digoxigenin (DIG) RNA labeling mix (Roche, Basel, Switzerland), 1 × T7 RNA polymerase buffer, 5 mM DTT, and 50 U T7 RNA polymerase (Takara, Kusatsu, Japan) were incubated at 37°C for 3 h for DIG labeling. After precipitation, the tubes containing DNase I (Roche, Basel, Switzerland) were incubated at 37°C for 30 min to remove all residual DNA. After precipitation, probes were eluted in a solution mix [5 × SSC, 50% deionized formamide, 50 μg/mL heparin, 50 μg/mL yeast t-RNA, 1 mM EDTA, 0.1% 3-[(3-cholamidopropyl)dimethylammonio]-1-propanesulfonate (CHAPS), 2 casein sodium salt, and 20% Tween 20 diluted in diethyl pyrocarbonate (DEPC, pH 7.5)] at a concentration of 1 μg/mL.

## 3. Results

### 3.1. Morphological changes during the development of the olfactory system

**FIGURE 1:**
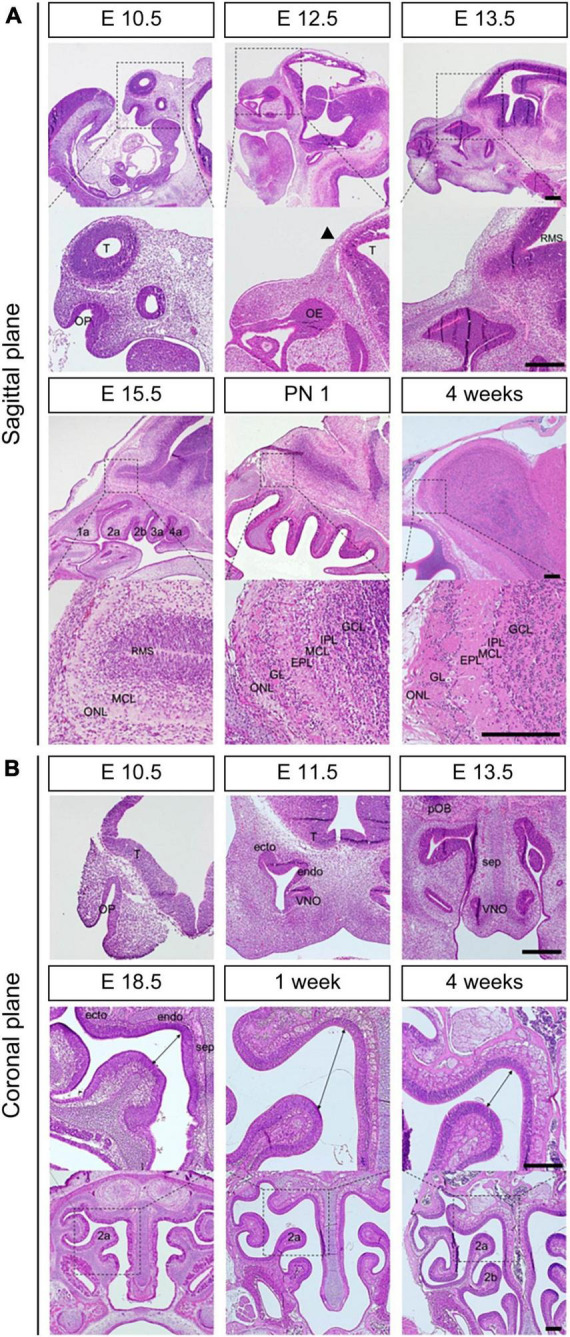
Morphological changes during the development of OE and OB. H&E staining **(A)** in the sagittal direction at E10.5, E12.5, E13.5, E15.5, PN 1, and 4 weeks and **(B)** in the coronal direction at E10.5, E11.5, E13.5, E18.5, 1 week, and 4 weeks. Black arrow in the sagittal direction at E12.5 indicates the tip of the telencephalon. The double arrows at E18.5, 1 week, and 4 weeks in the coronal direction indicate the distance between the each end of olfactory turbinate. T, telencephalon; OE, olfactory epithelium; RMS, rostral migratory stream; OP, olfactory pit; endo, endoturbinate; ecto, ectoturbinate; VNO, vomeronasal organ; MCL, mitral cell layer; ONL, olfactory nerve layer; GL, glomerular layer; EPL, external plexiform layer; IPL, internal plexiform layer; GCL, granule cell layer. Scale bar, 250 μm.

We examined the morphological changes in OE and OB from the prenatal to postnatal period. First, we focused on the OB and the interactive structure between OE and OB in the sagittal direction (Figure 1A). We found that the olfactory placode that later became OE was present on embryonic day 10.5 (E10.5). At E12.5, the tip of the telencephalon, where the putative OB (pOB) appeared, was formed. Later, a pOB appeared, with the narrowing and protrusion of the telencephalon at E13.5, which was accompanied by the narrowing of the entire ventricular zone in rostral migratory stream (RMS). At E15.5, the turbinate was divided into 1a, 2a, 2b, 3a, and 4a, as observed in adults. Simultaneously, the mitral cell layer (MCL) was formed between the RMS and olfactory nerve layer. At that time, the MCL was the first layer of the pOB. After birth, the glomeruli, where peri-glomerular neurons and olfactory nerves interacted, appeared, and the granular layer (GL) was formed in OB. Layers of OB, observed in adults, were formed on postnatal day 1 (PN 1).

Next, we analyzed the morphological changes in the coronal direction, focusing on the structure of the olfactory turbinate (Figure 1B). We found that organophosphates (OP), which first appeared at E10.5, were divided into endoturbinate, ectoturbinate, and VNO at E11.5. At E13.5, a septum was observed between both sides of the turbinate. In the period between E18.5 and 1 week, the volume ratio of olfactory cavity to olfactory epithelial layers increased. However, as the olfactory turbinate became more complex, the volume ratio of the olfactory cavity to olfactory epithelial layers decreased.

### 3.2. Changes in cellular composition of OE and olfactory cilia during the development

**FIGURE 2:**
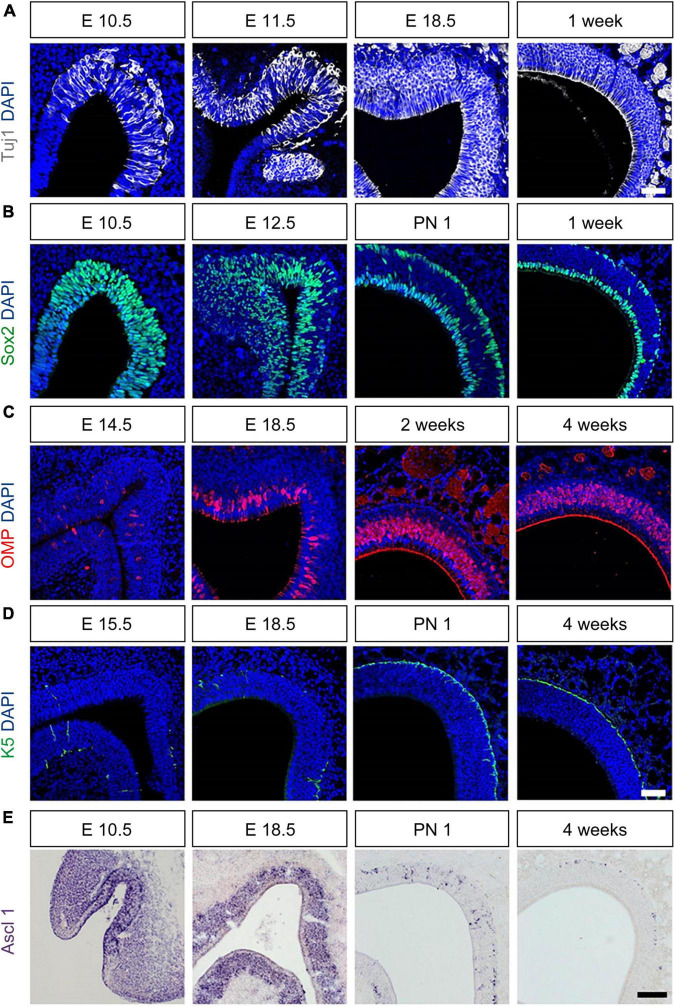
Molecular and cellular changes during the development of OE. All images were obtained in the coronal direction. **(A)** Immunostaining with an anti-Tuj1 antibody and DAPI at E1.5, E11.5, E18.5, and 1 week. **(B)** Immunostaining with an anti-Sox2 antibody and DAPI at E10.5, E12.5, E18.5, PN 1, and 1 week. **(C)** Immunostaining with anti-OMP antibody and DAPI at E14.5, E18.5, 2, and 4 weeks. **(D)** Immunostaining with an anti-K5 antibody and DAPI at E13.5, E18.5, PN 1, and 1 week. **(E)** *In situ* hybridization with an anti-Ascl probe at E10.5, E14.5, 2- and 4 weeks. Scale bar, 50 μm.

We investigated the change in cellular composition of OE according to the developmental stage from the prenatal to postnatal period (Figure 2). Cell-specific markers OMP, Tuj1, Sox2, ASCL1, and K5 were used to identify mature OSN (mOSN), immature OSN (iOSN), sustentacular cells (Sus), globose basal cells (GBC), and horizontal basal cells (HBC), respectively. The frequency of Tuj1+ neurons increased from E10.5 (Figure 2A). At E12.5, the Sox2- cell layer between the apical and basal layers appeared and the frequency of Sox- progenitors increased (Figure 2B). A monolayer of Sox2+ Sus appeared clearly on the apical side at 1 week (Figure 2B). K5+ HBCs were observed in some parts of the ectoturbinate at E13.5. However, K5+ HBCs in non-olfactory regions appeared earlier at E11.5 (Supplementary Figure 2B). The frequency of K5+ HBC increased over time (Figure 2C). The K5+ HBC layer on the basal side was clearly observed on PN1, similar to that in adults (Figure 2C). OMP expression was observed at E14.5, and the frequency of OMP+ neurons gradually increased (Figure 2D). After birth, the frequency of OMP+ cells increased up to 4 weeks (Figure 2D). The frequency of ASCL+ progenitors increased E10.5 onward (Figure 2E). However, a reduced frequency of ASCL1+ GBCs was observed in the PN 1 group (Figure 2E). At 1 week, a layer of ASCL11+ GBCs was evident on the basal side (Supplementary Figure 2C). In addition, the frequency of ASCL1+ GBCs in the endoturbinate decreased after birth (Figure 2E), but not in the ectoturbinate (Supplementary Figure 2C).

**FIGURE 3:**
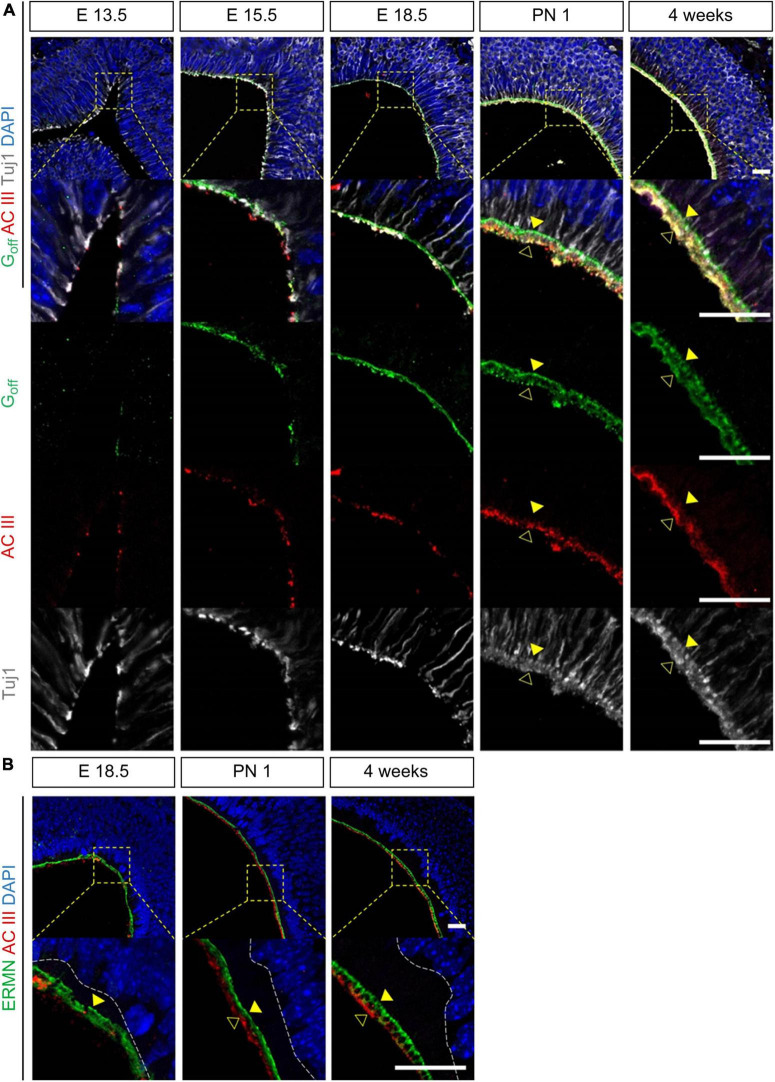
Molecular changes during the development of olfactory cilia. All images were obtained in the coronal direction. **(A)** Immunostaining with anti-G_olf_, anti-ACIII antibody, anti-Tuj1 antibody, and DAPI at E13.5, E15.5, E18.5, PN 1, and 1 week in the coronal direction. **(B)** Immunostaining with anti-ERMN, anti-ACIII antibody, and DAPI at E18.5, PN 1, and 4 weeks. Yellow solid arrows in panel **(A)** indicate Golf^+^ and ACIII^+^ cilia and yellow hollow arrows indicate only ACIII^+^ cilia. Yellow solid arrows in panel **(B)** indicate ERMN^+^ cilia. Scale bar, 20 μm.

Next, we analyzed the marker expression in olfactory cilia from the prenatal to postnatal period (Figure 3). We found that the markers for olfactory cilia, including Golf and ACIII, were expressed at E13.5 (Figure 3A), although Tuj1+ knobs and cilia appeared earlier, at E11.5 (Figure 2B). Before birth, only a few cilia expressed Golf or ACIII. At E18.5, ACIII+ cilia were few, but the cilia layer expressing Golf was linear. The two-layered olfactory cilia, observed in adults, formed after birth. Cilia that simultaneously expressed Golf and ACIII were rare. Golf and ACIII were co-expressed in the apical layers, whereas only Golf was expressed in the cilia of the basal layers. We analyzed both the olfactory and non-olfactory cilia after birth using the markers ACIII and ERMN, which are expressed in the cilia of Sus (Figure 3B). We found the cilia in basal layers expressing ERMN to be non-olfactory cilia, and those in apical layers expressing ACIII to be olfactory cilia. In addition, the thickness of the intermediate layer between the cilia and the nuclei of Sus increased after birth.

### 3.3. Projection of olfactory nerve fibers toward the telencephalon and layer-specific changes in OB during the olfactory development

**FIGURE 4:**
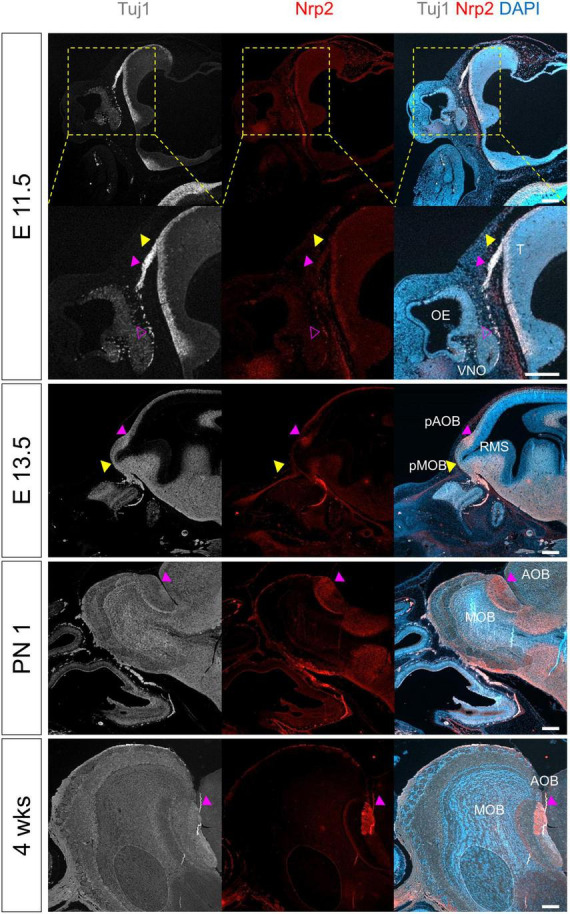
Morphological and molecular changes during the development of OB. All images were obtained in the sagittal direction. Immunostaining with anti-Nrp2 antibody, anti-Tuj1 antibody, and DAPI at E11.5, E13.5, PN 1, and 4 weeks. Yellow arrows indicate the tip of the telencephalon, putative olfactory bulb, or OB; magenta hollow arrows indicate Tuj1^+^ and Nrp2^+^ vomeronasal nerve fibers migrated from VNO; and magenta solid arrows indicate putative accessory olfactory bulb or accessory olfactory bulb. T, telencephalon; OE, olfactory epithelium; VNO, vomeronasal organ; RMS, rostral migratory stream; (p)MOB, putative main olfactory bulb; (p)AOB, putative accessory olfactory bulb. Scale bar, 200 μm.

Next, we examined the morphological changes in OB and the projection of olfactory axons to the telencephalon across the prenatal and postnatal periods (Figure 4). The cell-specific markers Tuj1, Nrp2, OMP, TH, and Reelin were used as representative markers for axons of the iOSN, nerves of subset of OSNs and VSNs, mOSN, periglomerular neurons, and mitral neurons, respectively. The first projection of olfactory nerves toward the forebrain (FB) was observed at E11.5. The olfactory nerves expressed Tuj1 and partially expressed Nrp2, which were also expressed in VSNs. The results suggested that projections of the olfactory and vomeronasal nerves are formed together. At E13.5, as the entire ventricular zone in RMS protruded toward the mid-portion of the olfactory nerve route, pOB was observed near the projection of the olfactory nerves at E11.5. At the same time, a putative AOB (pAOB) was formed at the dorsal region of pOB. After birth, morphology of the OB layers was similar to that observed in adults. Area of the Nrp2+ nerves in the AOB was reduced at 4 weeks.

**FIGURE 5:**
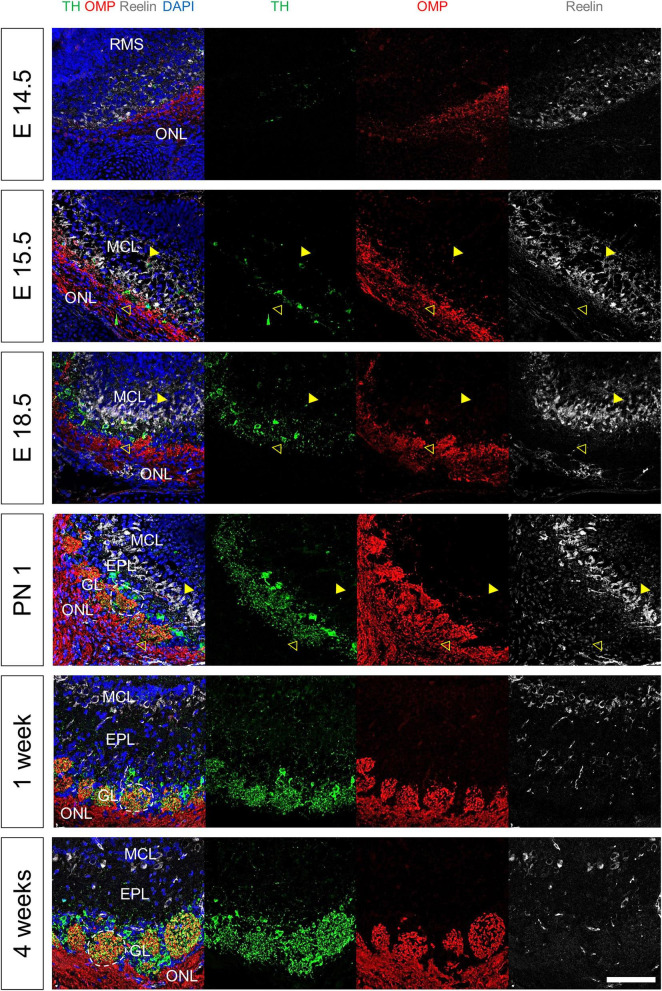
Molecular and cellular changes during the development of OB layers. All images were obtained in the sagittal direction. Immunostaining with anti-TH, anti-OMP, and anti-Reelin antibodies, and DAPI at E14.5, E15.5, E18.5, PN 1, and 4 weeks. Yellow solid arrows, yellow hollow arrows, and white lines indicate the MCL, GL, and glomerulus, respectively. Scale bar, 100 μm.

Finally, we analyzed the OB layers, focusing on the interactive structure between OE and OB (Figure 5). Reelin+ neurons were near the FB at E11.5 (Supplementary Figure 5A). TH+ and OMP+ neurons were also found near the pOB at E14.5. The TH + neurons were divided into two layers at E15.5. Later, one layer became the MCL and the other layer became the GL. We also observed that Reelin was expressed between the two layers. The number of TH+ neurons increased at E18.5. The glomeruli, wherein TH+ peri-glomerular neurons and OMP+ nerve interact, formed after birth.

## 4. Discussion

Since OE has a complex spatial organization (Saito et al., 1998; Duggan et al., 2008), study of OE at the 3D level is challenging. Therefore, we investigated the complex structure in both sagittal and coronal directions. In the sagittal direction of the olfactory structure, we observed an interactive structure between OE and OB, namely NFJ, which was hardly seen in the coronal direction. Thus, we investigated the histological changes that occur during the interaction between OE and OB in the sagittal direction. In addition, we were able to analyze the complex structure of OE, including the turbinate, septum, and VNO, in the coronal direction of the olfactory system. Overall, in the present study, we used both the sagittal and coronal directions to reveal the structure of OE.

**FIGURE 6:**
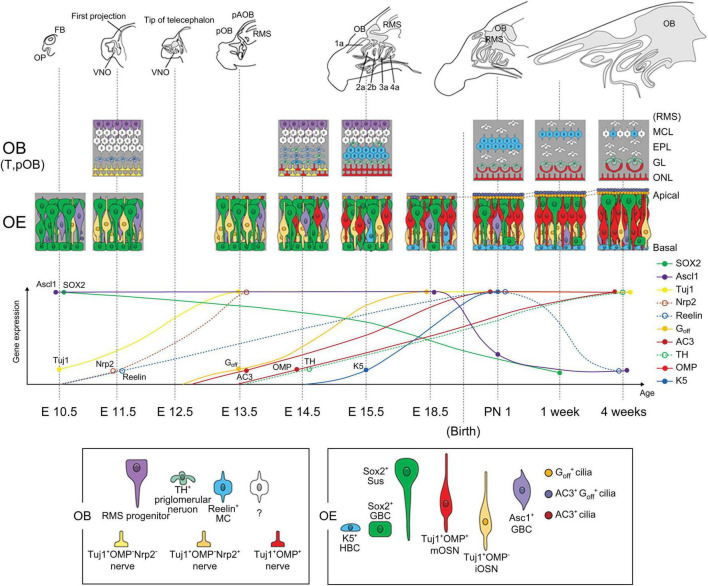
Schematic diagram of the developmental changes in olfactory system. The scheme presents the serial changes in morphology, cell composition, and gene expression with age.

Using spatial analysis according to the continuous developmental stages, we constructed a spatiotemporal map of olfactory development with the expression pattern spectrum of olfactory specific genes, as shown in Figure 6. The serial results of olfactory development are included in Supplementary Figures 1–5. The size and complexity of the olfactory system gradually increased over time. In the early developmental stages, the OE structure became more complex. Later, pOB including pMOB and pAOB were formed with the projection of neuronal fibers to the telencephalon and narrowing of the entire ventricular zone in RMS. At late developmental stages, the layers of OE and OB became multilayered and specified, accompanied by the differentiation of olfactory neurons. Remarkably, we found that OE differentiated and olfactory ciliary layers developed when air entered the nose after birth.

The current spatiotemporal analysis by using the specific genes allowed us to determine the time point of the first appearance of a specific structure and cell type. Because previous studies analyzed the olfactory development using general histological features like the size, the shape, and the signal density, this study using the specific genes makes the analysis of various cell types possible for the first time. Consistent with previous studies, we found that the first time point of nerve projection to the telencephalon was at E11.5 (Cuschieri and Bannister, 1975b; Watanabe et al., 2009; Miller et al., 2010; Forni and Wray, 2012). We also observed that the tip of the telencephalon appeared at E12.5, and pOB which consists of pMOB and pAOB and protrudes to the anterior was formed at E13.5. Previous studies had reported that the time point at which pOB first appears is E12.5, since they defined pOB as the protrusion of the telencephalon (Yoshihara et al., 2005). Here, pOB is defined as pOB with Nrp2^+^ pAOB-like OB, as shown in adults in the current study; we set that time at E13.5. Further, the present study provided a spectrum of the complexity of OE. OP was divided into three regions (endoturbinate, ectoturbinate, and VNO) at E11.5, and the morphology of olfactory turbinate (1a, 2a, 2b, 3a, and 4a), which was observed in adults, appeared at E15.5. The emergence and progression of olfactory cilia, HBC, and MCL were also analyzed. We observed the emergence of HBCs in olfactory regions at E13.5, whereas in another study, it was observed at E14 (Packard et al., 2011). We also observed the emergence of Reelin^+^ MCL in pOB at E15.5, whereas in other studies, they were observed at E16.5 and E18.5, respectively, (Bulfone et al., 1998; Faedo et al., 2002; Long et al., 2003; Yoshihara et al., 2005; Hirata et al., 2006; Cho et al., 2007; Shaker et al., 2012). These discrepancies in the timing of MCL emergence may have resulted from differences in markers and time points across different studies.

Furthermore, we speculated regarding a temporal correlation between the morphogenesis of structures in the olfactory system. We demonstrated when the first time is OB with AOB is formed, and why AOB was located at the dorsal region of OB. After the projection of neuron fibers and the formation of the tip of the telencephalon, pOB protruded with the narrowing of the entire ventricular zone in RMS, and pAOB was formed at the dorsal region of pOB. Since OB formation begins with the projection of fibers of OSNs or VSNs from OE, OE most likely affected OB formation. Also, previous studies reported that OE and OB show a link during development. The studies of the penetration of the pioneer axons to the basal lamina of the brain showed that the connectivity between OE and OB formed after penetration (Cuschieri and Bannister, 1975b; Watanabe et al., 2009). Also, the pioneer axons near the telencephalon induced neuronal differentiation at the anterior end of the telencephalon and the evagination of the OB (Gong and Shipley, 1995). Therefore, together with these studies, our results and speculations support the notion that the olfactory axon from OE influences OB morphogenesis. Moreover, there were previous studies that mutations in signaling molecules expressed in the olfactory system, like Gli3, ARX (aristaless related homeobox), and Fezf1 (Forebrain Embryonic Zinc Finger 1), cause OB agenesis (Yoshihara et al., 2005; Watanabe et al., 2009; Taroc et al., 2020). These investigations noted that several factors expressed in olfactory system regulate the OB morphogenesis.

On the other way of OE influence to OB during development, the differentiation of OE from iOSN to mOSN expressing G_olf_, ACIII, and OMP appeared after pOB formation. Based on this spatiotemporal sequence, OSN axons could be assumed to receive signals that induce the differentiation of mOSN from the interneurons of pOB. Previous studies had supported this assumption. Dopamine produced by TH^+^ dopaminergic neurons in OB is known to induce differentiation of the mOSN (Murrell and Hunter, 1999). Another study using mouse models of the neurodegenerative disease reported that a decrease in glomerular dopaminergic neurons resulted in a decrease in mOSNs in OE (Son et al., 2021). In addition, the MCL and GL develop interactively, wherein olfactory axons and OB neurons affect each other. Also, a distinct olfactory receptor expressed in each OSN mediated the anterior-posterior projection to OB and glomeruli segregation in OB in both directions (Takeuchi and Sakano, 2014). These investigations noted that several factors expressed in the olfactory system regulate the OB morphogenesis. Therefore, although OE is independently generated during the early developmental stage, before the formation of axonal connectivity between OE and OB by penetration of the pioneer axons to the telencephalon, the olfactory system is developed by the cooperation of OE and OB in the late developmental stage.

Interestingly, we observed changes in the OE following exposure to air before and after birth. Since olfactory signals are generated by the binding of odorants present in the air to the olfactory cilia, exposure to air could stimulate the development of OE. In fact, the apical and basal layers of olfactory cilia became olfactory and respiratory cilia, respectively, after birth. This finding indicated that olfactory cilia immediately after birth may be ready to receive odorant stimuli, which may be sufficient to generate olfactory signals. In addition, the intermediate layer between the olfactory cilia and nuclei of the Sus cells gradually increased after birth. Electron microscopic analysis of OE revealed the presence of mitochondria in the Sus cells and OSNs in the intermediate layer (Falk et al., 2015). Therefore, mitochondria number and their space in this intermediate layer may be assumed to increase following exposure to air. This clarified layer of cilia and the increase of the energy metabolism by mitochondria mean the increase of the capability of olfactory signaling. A previous study that mitochondria in the mOSN play a role in olfactory signaling (Fluegge et al., 2012) may support this notion. Moreover, given that the Sus cells which are located at the apical region in OE protect OSNs and other cells in OE, the increase of mitochondria number in Sus cells means that the protectable competence by the energy metabolism of mitochondria increased against the environmental change. To prove this theory, further studies on the role of mitochondria in Sus cells and OSNs are needed. At the level of OE, the frequency of ASCL1^+^ progenitors drastically decreased after birth, whereas the frequency of K5^+^ HBC and OMP^+^ mOSN gradually increased. The findings implied that encounters with air facilitate not only the differentiation of OE but also the regeneration capability of HBCs (Yu and Wu, 2017; Sokpor et al., 2018). Together with the increase of protective ability at the apical region of OE, the regeneration ability at the basal region of OE means the increase of the protective ability in OE. The investigations supporting this notion are needed further. At the level of OB, the size of GL, in which olfactory axons are combined with glomerular neurons, increased. This phenomenon correlated with the increase in OSNs. However, whether the length or the number of OSNs induces the increase in the size of each glomerulus should be studied further. To the best of our knowledge, our study is the first to report these events related to olfactory cilia following the exposure to air.

However, this investigation had some limitations. First, we focused on the endoturbinate, which is present in the olfactory structure during development, although the structure of OE is more complex. Given this complexity in OE, the study of the anterior to the posterior on the ectoturbinate is needed to comprehend the overall OE structure. Second, we used several markers to analyze several cell types of both OE and OB. There are various markers of the olfactory lineage of OSN, especially those of GBC (Fletcher et al., 2017; Yu and Wu, 2017; Sokpor et al., 2018). Therefore, it should be studied what genes are expressed in distinct GBC at each developmental stage to figure out the gene expression pattern in embryonic neurogenesis. Third, further studies using a higher magnification method would be required to examine the ultrastructure, such as centrioles in the olfactory knob and tubulins in the olfactory cilia. These limitations need to be addressed for a better understanding of the fine structures in the olfactory system during development. Nevertheless, our study is sufficient to understand the olfactory system during development.

In summary, we demonstrated the spatiotemporal dynamics of mouse olfactory system during development by analyzing the changes in morphology and cellular composition from prenatal to postnatal period. Recent investigations using specific methods can study the expression and the role of the specific genes better than before. This study provides an overview of olfactory development with the expression pattern of each specific gene, the sequential change between developmental events, especially after birth, as the development stage progress, and the correlation between OE and OB during development. To sum up, the present study provides several important insights into the understanding of defects during olfactory development.

## Data availability statement

The datasets presented in this study can be found in online repositories. The names of the repository/repositories and accession number(s) can be found below: https://www.ncbi.nlm.nih.gov/, NM_008553.5.

## Ethics statement

This animal study was reviewed and approved by the Yonsei Medical Center Animal Research Guidelines (IACUC No. 2020-0030).

## Author contributions

B-RK: conceptualization, methodology, analysis, and writing. M-SR: conceptualization and writing. H-JC, J-HY, and C-HK: investigation, supervision, and review editing. All authors read and agreed to the submitted version of the manuscript.
