# Supplementary material for: Spatiotemporal dynamics of the development of mouse olfactory system from prenatal to postnatal period
Source: Front Neuroanat. 2023 Apr 11;17:1157224. doi: 10.3389/fnana.2023.1157224 (PMC10126376; doi:10.3389/fnana.2023.1157224)
Supplement: Supplementary file 1 [file Table_1.DOCX]

Supplementary Material

Spatiotemporal Dynamics of the Development of Mouse Olfactory System from Prenatal to Postnatal Period

**Bo-Ra Kim, Min-Seok Rha, Hyung-Ju Cho, Joo-Heon Yoon, Chang-Hoon Kim***

*** Correspondence:** Chang-Hoon Kim: entman@yuhs.ac


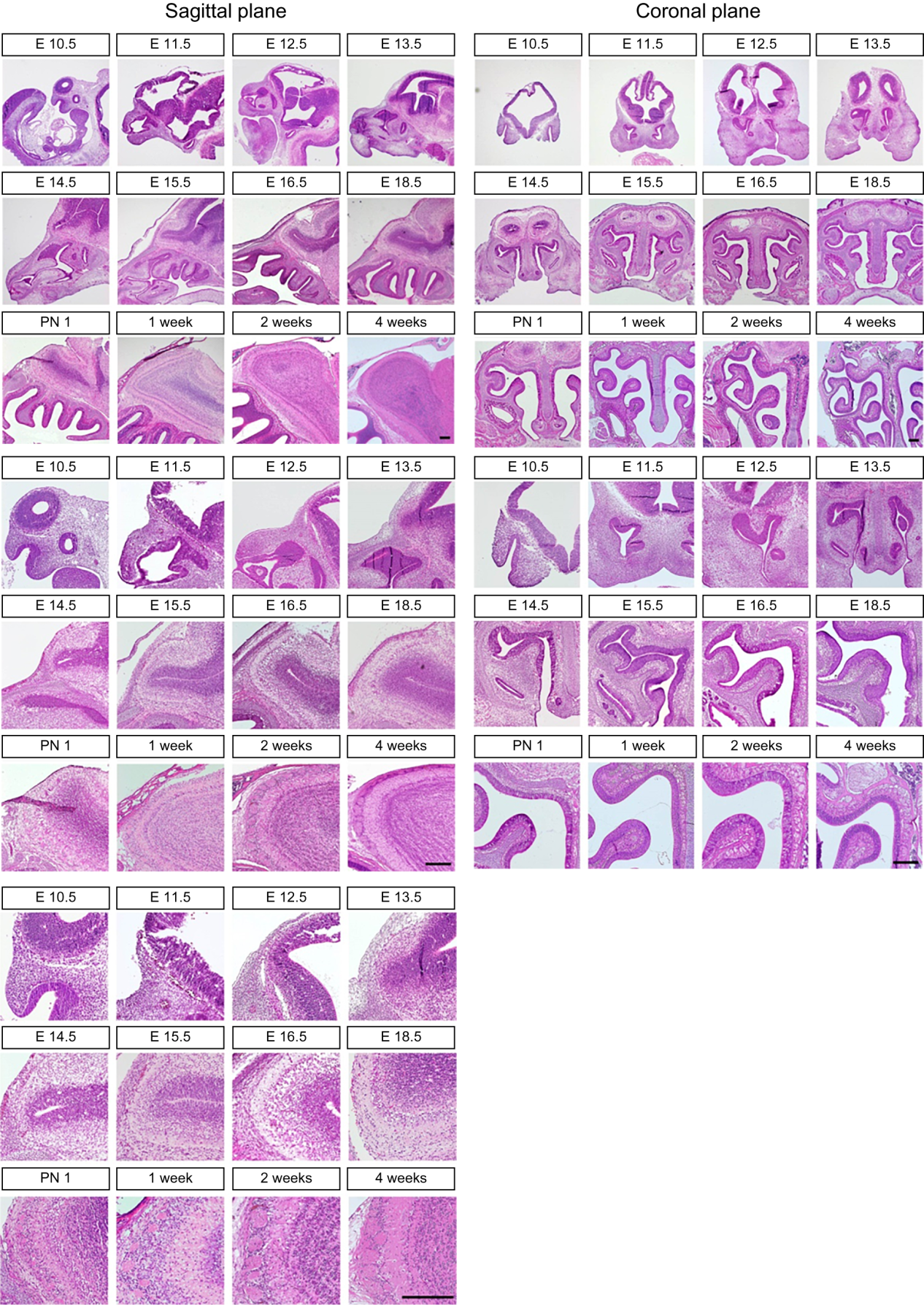


**Supplementary Figure 1.** Serial changes in morphology during development in OE and OB. H&E staining in sagittal and coronal directions. Scale bar, 250 μm.

**
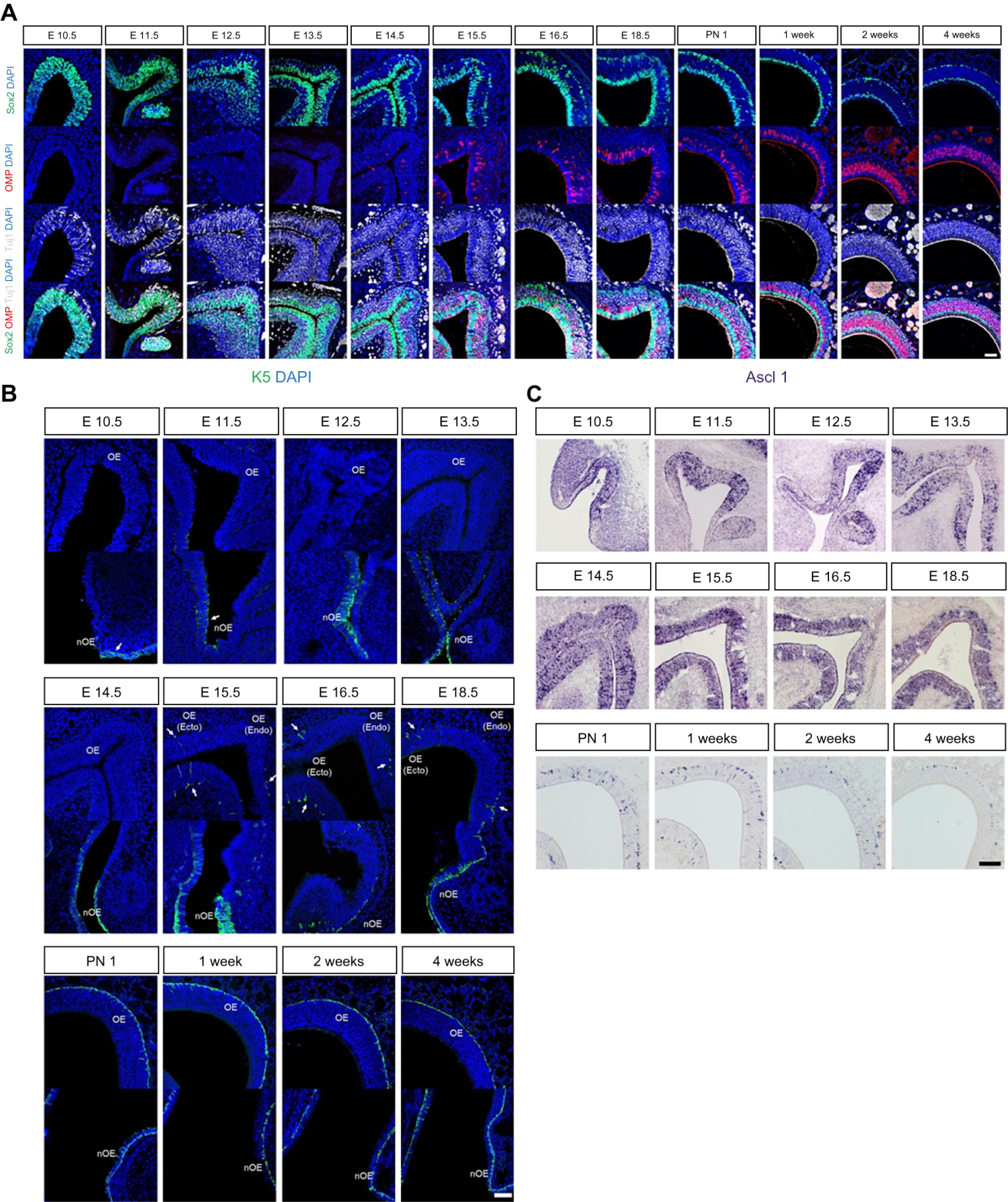
**

**Supplementary Figure 2.** Serial changes in morphology during OE development. All images are in coronal direction. (**A**) Immunostaining with anti-Sox2, anti-OMP, and anti-Tuj1 antibodies, DAPI, and merged images. (**B**) Immunostaining with anti-K5 antibody and DAPI in olfactory and non-olfactory regions. (**C**) *In situ* hybridization with anti-Ascl probe. Scale bar, 50μm. OE, olfactory epithelium; Endo, endo-turbinate; Ecto, ecto-turbinate; nOE, non-olfactory epithelium.

**
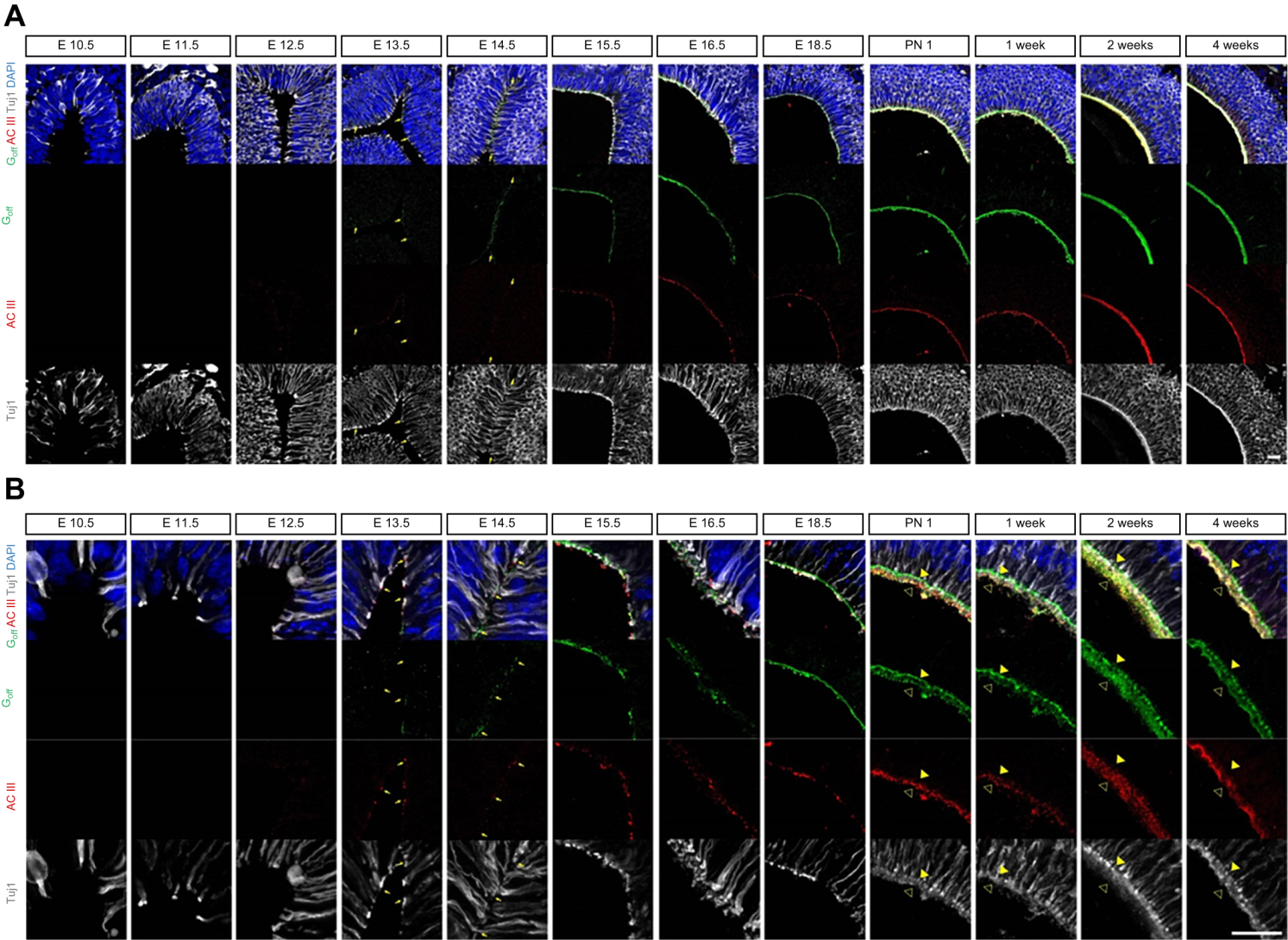
**

**Supplementary Figure 3.** Serial changes in morphology during development in olfactory cilia.

All images are in coronal direction. (**A**) Immunostaining with anti-G_olf_, anti-ACⅢ, and anti-Tuj1 antibodies and DAPI. (**B**) Higher magnification of panel (**A**). Yellow single arrows in E13.5 and E14.5 indicate the expression of each marker. Yellow full arrows in PN 1, 1-, 2-, and 4 weeks indicate G_olf_^+^ and ACⅢ^+^ cilia. Yellow hollow arrows in PN 1, 1-, 2-, and 4 weeks indicate only ACⅢ^+^ cilia. Scale bar, 20 μm.

**
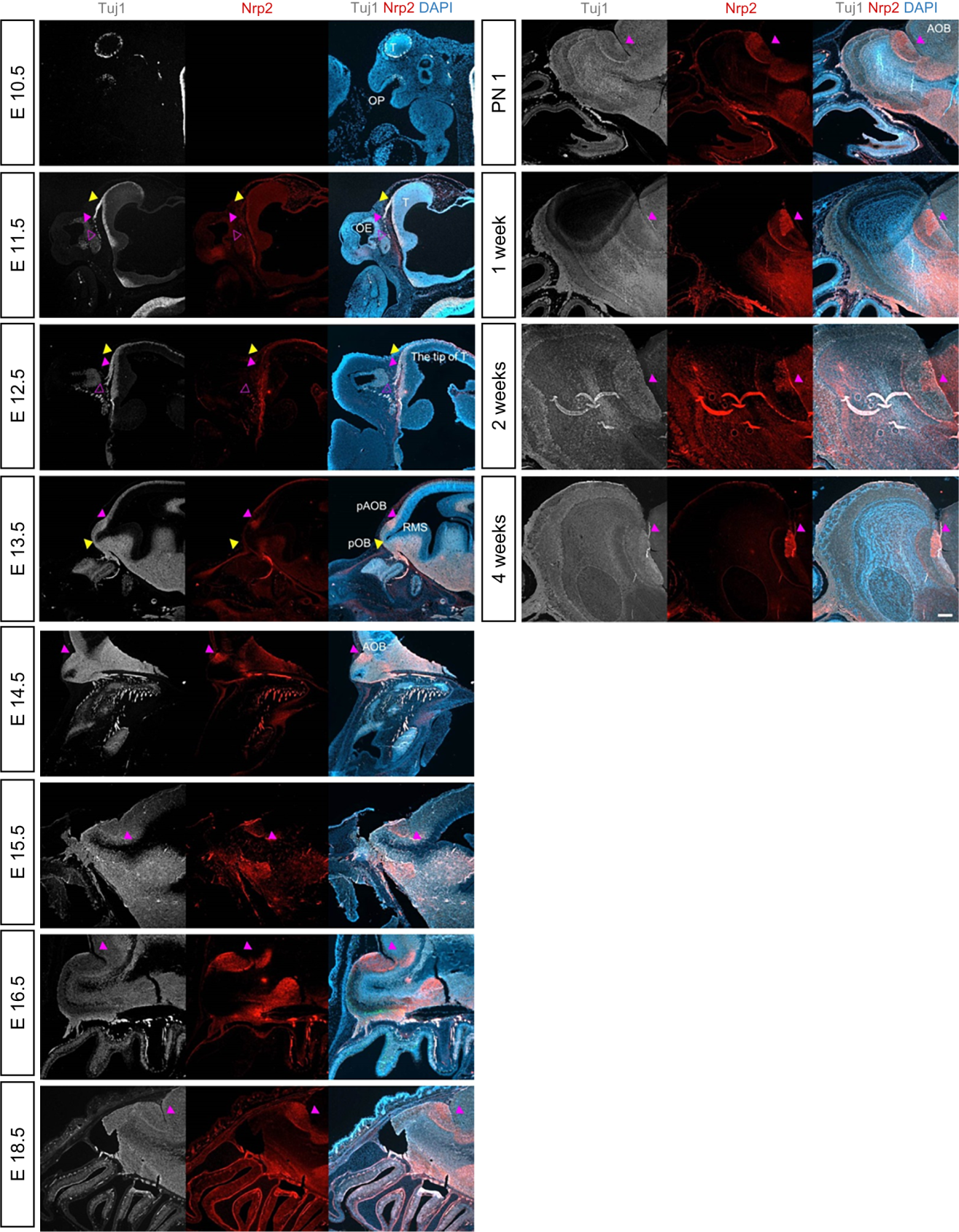
**

**Supplementary Figure 4.** Serial changes in morphology and markers during development in OB. All images are in sagittal direction. Immunostaining with anti-Nrp2 and anti-Tuj1 antibodies, and DAPI. Yellow arrows indicate the tip of telencephalon, putative olfactory bulb or OB, magenta hollow arrows indicate Tuj1^+^ and Nrp2^+^ olfactory nerve fiber, and magenta full arrows indicate putative accessory olfactory bulb or accessory olfactory bulb. Scale bar, 200 μm. T, telencephalon; OP, olfactory pit; (p)OB, putative olfactory bulb; (p)AOB, putative accessory olfactory bulb.

**
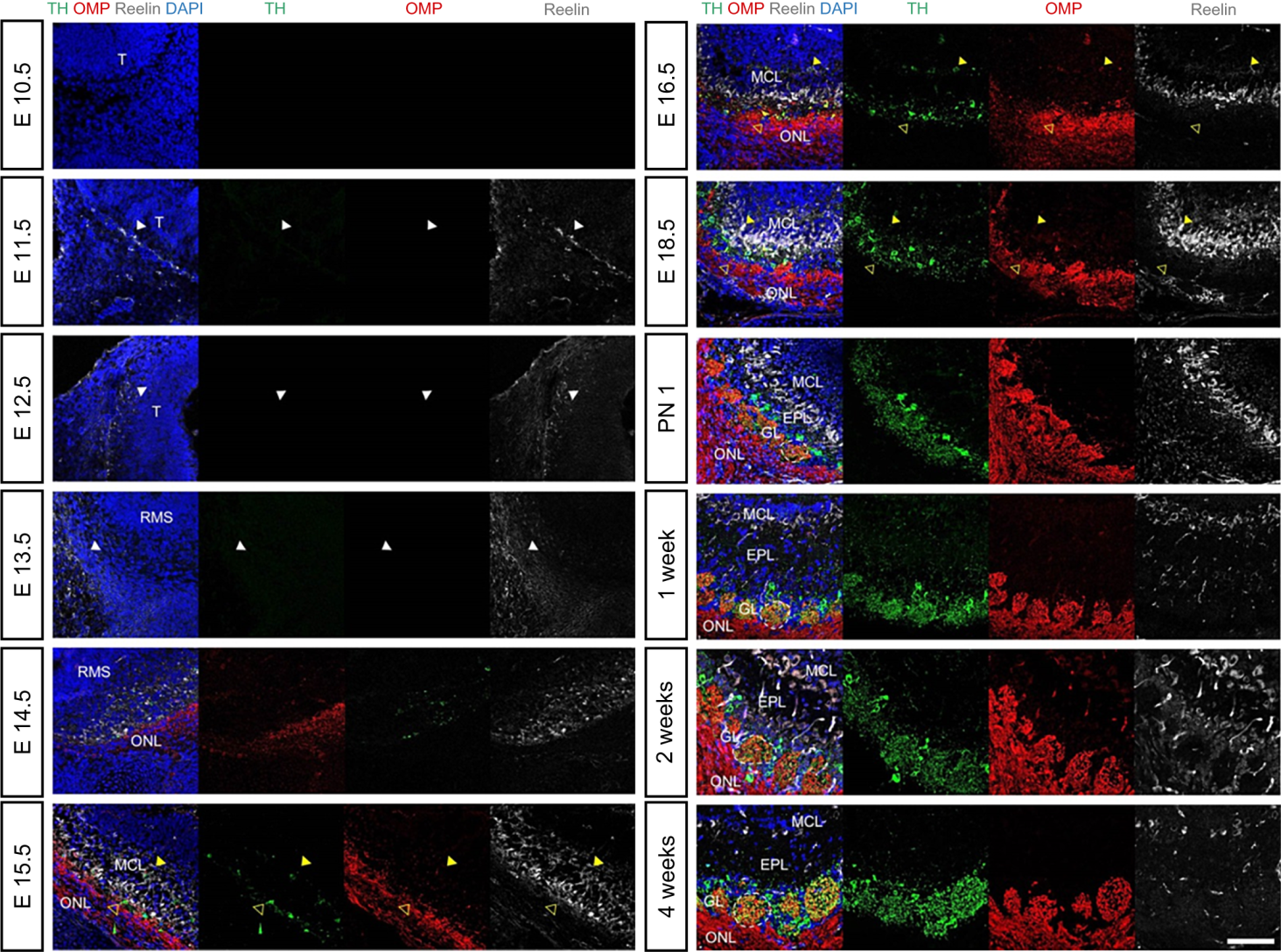
**

**Supplementary Figure 5.** Serial changes in morphology and cell composition during development in olfactory bulb layers. All images are in sagittal direction. Immunostaining with anti-TH, anti-OMP, and anti-Reelin antibodies, and DAPI. Yellow full arrows indicate MCL, yellow hollow arrows indicate GL, and white lines indicate glomerulus. Scale bar, 100μm. T, telencephalon; RMS, rostral migratory stream; ONL, olfactory nerve layer; MCL, mitral cell layer; EPL, external plexiform layer; GL, glomerular layer.
